# High-flow nasal cannula oxygen reduced hypoxemia in patients undergoing gastroscopy under general anesthesia at ultra-high altitude: a randomized controlled trial

**DOI:** 10.1186/s12871-024-02568-9

**Published:** 2024-05-27

**Authors:** Dunzhu Zhaxi, Deji Ci, Xiang Quan, Ciren Laba

**Affiliations:** 1 Department of Anesthesiology, Tibet Autonomous Region People’s Hospital, Lhasa, Tibet China; 2 Department of Anesthesiology, Peking Union Medical College Hospital, Chinese Academy of Medical Sciences and Peking Union Medical College, Beijing, China

**Keywords:** High-flow nasal cannula (HFNC), Gastroscopy, High altitudes, Hypoxemia, Anesthesia

## Abstract

**Background:**

Hypoxemia can occur in people at ultra-high altitude (above 3500 m) even at rest, and patients undergoing gastroscopy under general anesthesia have higher risk of hypoxemia. Supplementary oxygen via standard nasal cannula (SNC) is the standard of care for most patients who undergo gastroscopy under general anesthesia, which provides oxygen flow up to 15 L/min. High-flow nasal cannula (HFNC) could deliver oxygen at a rate up to 60 L/min, which is recommended by the American Society of Anesthesiologists Practice Guidelines. We speculated that the benefit with HFNC is more prominent in high-altitude areas, and aimed to compare the incidence of hypoxemia during gastroscopy under general anesthesia at ultra-high altitude with oxygen supply via either HFNC or SNC.

**Methods:**

The trial was registered at at Chinese Clinical Trial Registry (ChiCTR2100045513; date of registration on 18/04/2021). Adult patients undergoing gastroscopy with anesthesia (estimated duration of anesthesia at ≥ 15 min) were randomized at a 1:1 ratio to receive HFNC oxygen or SNC oxygen. The primary outcome was hypoxemia (SpO_2_ < 90% for any duration). Secondary outcomes included severe hypoxemia (SpO_2_ < 75% for any duration or SpO_2_ < 90% but ≥ 75% for ≥ 60 s) and hypotension, as defined by reduction of mean arterial blood pressure by ≥ 25% from the baseline.

**Results:**

A total of 262 patients were enrolled: 129 in the HFNC group and 133 in the SNC group. All patients received the designated intervention. Student’s t-test, Mann-Whitney U test and χ^2^ test were employed in the study. The rate of hypoxemia was 9.3% (12/129) in the HFNC group versus 36.8% (49/133) in the SNC group [risk ratio (95% confidence interval): 0.25(0.14–0.45); *P* < 0.001). The HFNC group also had lower rate of severe hypoxemia [0.0% (0/129) versus 11.3% (15/133); risk ratio (95% confidence interval): 0.03(0.00-0.55); *P* < 0.001, respectively]. The rate of hypotension did not differ between the 2 groups [22.5% (29/129) in HFNC group versus 21.1% (28/133) in SNC group; risk ratio (95% confidence interval): 1.07(0.67–1.69) ; *P* = 0.779].

**Conclusion:**

HFNC oxygen reduced the incidence of hypoxemia during anesthesia in adult patients undergoing gastroscopy at ultra-high altitude.

## Introduction

Gastroscopy is typically performed under general anesthesia to provide optimal procedural conditions and to minimize patient discomfort [1, 2]. If conducted properly, complications associated with anesthesia (e.g., hypoxemia and hypotension) are uncommon [3, 4], but may occur at much higher rate in high-altitude areas due to low partial pressure of oxygen [5].

Supplementary oxygen via standard nasal cannula (SNC) is the standard of care for most patients who undergo gastroscopy under general anaesthesia [1]. SNC provides oxygen flow up to 15 L/min and an inspired oxygen concentration in the distal airways at 30-40% [6]. Higher inspired oxygen concentrations are not possible with SNC because of air mixing and dilution with carbon dioxide from dead space [6, 7].

High-flow nasal cannula (HFNC) could deliver 100% humidified and heated oxygen at a rate up to 60 L/min [8–10]. Based on the reduced hypoxemia with HFNC in critically ill patients with acute respiratory failure [11–13], HFNC is recommended by the American Society of Anesthesiologists Practice Guidelines in patients with respiratory suppression or apnea, and for unanticipated difficult airways [14]. HFNC oxygen also reduces airway obstruction by increasing distending pressure in the upper airway [15].

Mazzeffi et al. [16] and Lin et al. [17] showed that HFNC reduces the incidence of hypoxemia in patients undergoing gastroscopy with anesthesia at sea level, but no clinical trials have been conducted at ultra-high altitude. We speculated that the benefit with HFNC is more prominent in high-altitude areas, and conducted a randomized controlled trial to test such a hypothesis.

## Methods

### Trial design and oversight

This parallel-group randomized controlled trial was conducted at the Tibet Autonomous Region People’s Hospital (Lhasa, Tibet, China; altitude: 3650 m above the sea level). The trial protocol was approved by the institutional review board of Tibet Autonomous Region People’s Hospital (ME-TBHP-20-KJ-032) in accordance with the Declaration of Helsinki, and was registered at http://www.chictr.org.cn (ChiCTR2100045513) on 18/04/2021. Written informed consent was obtained from all participants prior to enrollment. Writing of the manuscript followed the Consolidated Standards of Reporting Trials (CONSORT) reporting guidelines.

Adult patients (≥ 18 years of age) scheduled for elective gastroscopy under general anesthesia and with an anticipated anesthesia duration at ≥ 15 min were eligible. Patients with high risk of reflux aspiration (pregnant women, gastroesophageal reflux) and severe nasal obstruction were excluded. Eligible patients were randomized at a 1:1 ratio to receive either HFNC oxygen using a OptiFlow THRIVE device (Fisher and Paykel Healthcare; Panmure, Auckland, New Zealand) or SNC oxygen. Randomization sequence was generated by a statistician not involved in this trial otherwise. Concealment was conducted using sealed opaque envelopes. Patients, physicians, and outcome assessors were not blinded to group assignment.

After the patients underwent hemodynamic monitoring and pulse oximetry monitoring (IntelliVue MP70 M8007A, Philips Medical Systems, Boeblingen, Germany), oxygen supplementation (HFNC oxygen at 20 L/min, 37 °C, oxygen concentration 100% or SNC oxygen at 6 L/min) started at 2 min prior to intravenous injection of 1-mg/kg propofol. Sedation level was monitored using the Observer’s Assessment of Alertness/Sedation (OAA/S) scale [18] every 2 min, and maintained at OAA/S of 1 (i.e. not responding to mild prodding or shaking) throughout the procedure by incremental boluses of 20 mg propofol. After administration of propofol, oxygen flow was increased to 40 L/min in the HFNC group and 10 L/min in the SNC group.

In cases of hypoxemia (SpO_2_ < 90%), jaw-thrust maneuver was conducted to maintain the airway. Mask ventilation was used when severe hypoxemia (SpO_2_ < 75% for any duration, or SpO_2_ < 90% but ≥ 75% for ≥ 60 s). Tracheal intubation was performed at the discretion of attending anesthesiologists. The study was performed according to the protocol.

Blood pressure, heart rate, pulse oximetry and end-tidal carbon dioxide were recorded at the baseline and every 2 min during anesthesia. Hypotension was managed at the discretion of the attending anesthesiologists. After completion of the procedure, patients were monitored for at least 20 min. Throughout the entire study period, epistaxis should be observed, which is a potential complication of using nasal cannula.

## Outcome measures

The primary end point was hypoxemia (SpO_2_ < 90% for any duration) during anesthesia (i.e., from propofol injection to the time when the patient’s responding after his/her name was called loudly after procedure) [19]. Secondary outcomes included severe hypoxemia (SpO_2_ < 75% for any duration or SpO_2_ < 90% but ≥ 75% for ≥ 60 s) and hypotension, as defined by reduction of mean arterial blood pressure by ≥ 25% from the baseline. All outcome measures were recorded in real time by an investigator who was not involved in patient care otherwise, and verified using video recording.

### Statistical analysis

Sample size requirement was estimated based on: (1) the rate of hypoxemia at 35% in the SNC group (unpublished preliminary data) and relative reduction of hypoxemia by 50% (to an absolute rate of 17.5%) in the HFNC group; (2) 2-sided α of 0.05 and 90% power. The calculation yielded 262 patients (131 in each group).

Continuous variables were compared between the 2 groups using Student’s t-test and presented as mean value ± standard deviation upon normal distribution, and using Mann-Whitney U test and presented as median and interquartile range (IQR) otherwise. Categorical variables were analyzed using χ^2^ test and presented as number and percentage. *P* < 0.05 was considered statistically significant. All statistical analyses were conducted using IBM SPSS Statistics (version 25.0; IBM Corp., Armonk, NY, USA). Risk ratios with 95% confidence intervals were reported for study outcomes.

## Results

### Patients

**Fig. 1:**
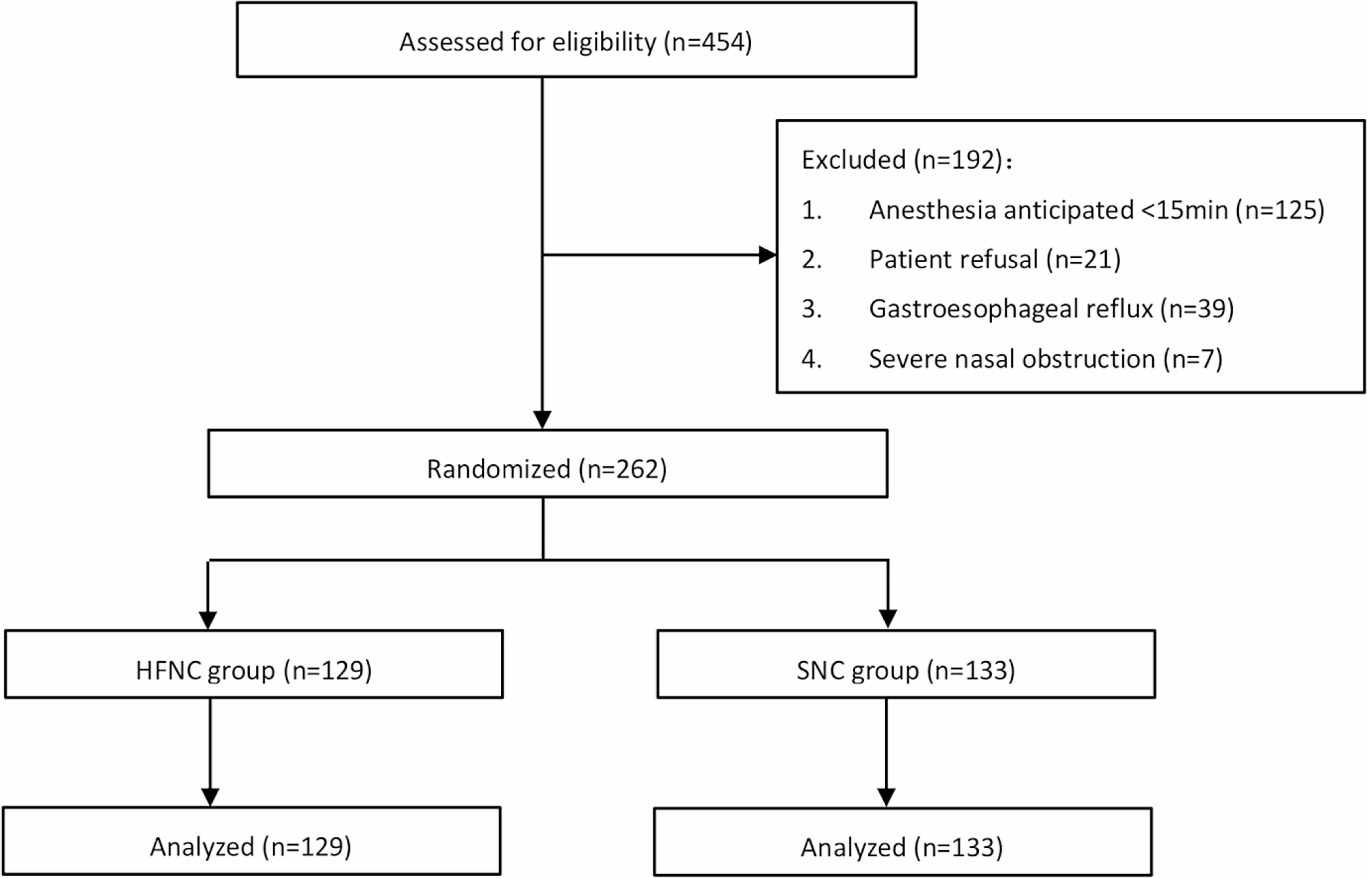
Patient flow through the trial

A total of 454 patients were screened during a period from April 2021 to July 2021 (Fig. 1); 262 patients were randomized (129 and 133 in the HFNC and SNC control groups, respectively). The study ended because a sufficient number of subjects were included. Demographic and baseline characteristics were generally balanced between the 2 groups (Table 1).

**Table 1:** Demographic and baseline characteristics of patients

Characteristics	HFNC (*n* = 129)	SNC (*n* = 133)	*P* value
Age (y)	48.6 ± 5.2	49.1 ± 4.8	0.419
Male sex, n(%)	62(48.1%)	59(44.4%)	0.548
Body mass index (kg/m^2^)	24.1 ± 3.1	23.8 ± 2.9	0.419
Local residence ≥ 3 months, n(%)	125(96.8%)	128(96.2%)	0.770
ASA grade, n(%)			0.660
I	28(21.7%)	32(24.1%)	
II	88(68.2%)	84(63.2)	
III	13(10.1%)	17(12.8%)	
Comorbidity, n(%)			
Hypertension	51(39.5%)	45(33.8%)	0.338
Coronary artery disease	12(9.3%)	8(6.0%)	0.316
Diabetes	10(7.8%)	15(11.3%)	0.331
Obstructive sleep apnea	19(14.7%)	14(10.5%)	0.305
Asthma	5(3.9%)	3(2.3%)	0.446
COPD	3(2.3%)	2(1.5%)	0.680
Interstitial lung disease	1(0.8%)	2(1.5%)	>0.999
Hemoglobin concentration (g/L)	160.2 ± 23.6	158.1 ± 25.9	0.494
Baseline vital signs			
SpO_2_ at room air (%)	90.3 ± 2.3	90.4 ± 2.7	0.748
Mean blood pressure (mmHg)	99.3 ± 18.8	97.1 ± 19.3	0.351
EtCO_2_ (mmHg)	35.8 ± 1.2	36.1 ± 1.6	0.088
Anesthesia characteristics			
Gastroscopy time (min)	13.3 (10.5–18.8)	12.5 (10.0–18.0)	0.220
Anesthesia time (min)	18.4(15.3–25.8)	18.1(15.1–24.1)	0.577
Total propofol dosage (mg)	223.3(133.5-310.7)	210.1(158.6-303.9)	0.080

Data are expressed as mean ± SD, median (IQR), or number (percentage)

ASA, American Society of Anesthesiology classification of physical status; COPD, chronic obstructive pulmonary disease; EtCO_2_, end-tidal carbon dioxide; HFNC, high-flow nasal cannula; SNC, standard nasal cannula; SpO_2_, peripheral oxygen saturation

### Outcomes

The rate of hypoxemia was 9.3% (12/129) in the HFNC group versus 36.8% (49/133) in the SNC group (*P* < 0.001; Table 2). The HFNC group also had lower rate of severe hypoxemia (0% versus 11.3%; *P* < 0.001; Table 2). The 2 groups did not differ in the rate of hypotension. Tracheal intubation was not performed in either group. Epistaxis was not observed in any patient in either group.

**Table 2:** Outcomes in the 2 groups

Outcome	HFNC (*n* = 129)	SNC (*n* = 133)	Risk ratio (95% CI)	*P* value
Hypoxia, n(%)	12(9.3%)	49(36.8%)	0.25(0.14–0.45)	< 0.001
Severe hypoxia, n(%)	0(0.0%)	15(11.3%)	0.03(0.00-0.55)	< 0.001
Hypotension, n(%)	29(22.5%)	28(21.1%)	1.07(0.67–1.69)	0.779

Data are presented as number (percentage). HFNC, high-flow nasal cannula; SNC, standard nasal cannula; CI, confidence interval

## Discussion

The current trial showed a robust reduction in the rate of hypoxemia in the HFNC group (9.3%) as compared to that in the SNC group (36.8%). The rate of severe hypoxemia was also lower in the HFNC group (0.0% versus 11.3% in the SNC control group). Hypotension did not differ between the 2 groups.

In a previous trial in patients undergoing gastrointestinal endoscopy with propofol (OAA/S scale maintained at < 3) at ultra-high altitude (3650 m) [20], supraglottic jet oxygenation and ventilation (SJOV) decreased the rate of moderate hypoxemia (SpO_2_ < 90% but ≥ 75% for < 60 s) during gastrointestinal endoscopy from 47.2% (17/36) in the SNC group to 8.3% (3/36). The rate of severe hypoxemia was decreased from 25.0% (9/36) to 0.0% (0/36). SJOV-related complications included nasal bleeding (8.3%), pharyngalgia (2.8%) and xerostomia (2.8%). In the current study, we only recruited patients undergoing gastroscopy and OAA/S was maintained of 1 by propofol. The results demonstrated robust reduction in the rate of hypoxemia (from 36.8 to 9.3%) as well as severe hypoxemia (from 11.3 to 0.0%) in the HFNC group. No HFNC-related complications were observed, confirming similar efficacy of HFNC to SJOV but less side adverse events.

The rate of hypoxemia in the SNC group in the current study was much higher than reported by a large trial by Lin et al. that compared HFNC oxygen with SNC oxygen in adult patients undergoing gastroscopy under propofol anesthesia at the sea level [17]. Such a discrepancy could be reasonably attributed to difference in altitude. In addition, the mean procedure time was higher in the current study (13 min versus 5 min in the Lin et al. trial). Another notable difference between the 2 studies is the lower HFNC flow rate in the current study (40 L/min versus 60 L/min in the Lin et al. trial). Such a rate in the current study represents the maximum achievable rate due to the ultra-high altitude. The efficacy of HFNC is apparently dependent on the flow rate since Mazzeffi et al. [16] reported only a modest reduction in the incidence of hypoxemia (from 33.1 to 21.2%) with 20 L/min HFNC in patients undergoing gastroscopy at the sea level.

Lower oxygen partial pressure at high altitude causes alveolar hypoxia and hypoxemia. Also, the temperature and humidity are lower compared to the sea level [21]. The benefits of HFNC include 100% FiO_2_ (fraction of inspired oxygen), lower positive end expiratory pressure that decreases alveolar collapse, and less stimulation to the airway [22]. In the present study, FiO_2_ was set at 100% in the HFNC group. Because of the high flow rate, we suspect the actual inspired oxygen concentration in patients in the HFNC group was close to 100% [8]. In contrast, 10 L/minute oxygen via SNC has been shown to produce < 80% FiO_2_ at the sea level [7, 8]. At the ultra-high altitude in the current study (3650 m), the actual FiO_2_ could be even less. HFNC also produces lower positive airway pressure, mainly determined by the flow rate [23]. In the present study, the oxygen flow rate was 40 L/min, resulting in a calculated positive airway pressure of 1.3 cmH_2_O in an open mouth during gastroscopy [23], which increases the end-expiratory lung volume.

A recent study conducted at 2600-m altitude reported 75% success (as defined by not requiring invasive mechanical ventilation) with HFNC treatment in ICU patients with hypoxemic respiratory failure [24]. Results of the current study added much-needed support for the benefits of HFNC in managing patients in high-altitude areas.

This trial has several limitations. First, we did not measure EtCO_2_ (end tidal carbon dioxide) after oxygen supply due to technical difficulty (interference of the measurement by the high flow of oxygen). Second, as a single-center trial, whether the results are applicable to the general practice setting at high altitude requires further confirmation. Third, we did not conduct a cost-benefit analysis, since HFNC has not yet been covered by medical insurance and can only be self funded, it is clear that the cost of the HFNC is definitely higher than the SNC. If HFNC can be coverd by the medical insurance in the future, the cost will be greatly reduced.

In summary, HFNC oxygen therapy reduced the incidence of hypoxemia, and particularly severe hypoxemia in adult patients undergoing gastroscopy under propofol anesthesia at ultra-high altitude.

## Data Availability

Data is provided within the manuscript.

## Declarations

### Clinical trial registration statement

The trial is registered at Chinese Clinical Trial Registry (ChiCTR2100045513; principal investigator Xiang Quan, date of registration on 18/04/2021).

## References

[1] Early DS, Lightdale JR, Vargo JJ, Acosta RD, Chandrasekhara V, Chathadi KV, Evans JA, Fisher DA, Fonkalsrud L, Hwang JH, Khashab MA, Muthusamy VR, Pasha SF, Saltzman JR, Shergill AK, Cash BD, DeWitt JM, ASGE Standards of Practice Committee (2018). Guidelines for sedation and anesthesia in GI endoscopy. Gastrointest Endosc.

[2] Goudra B (2019). Big sleep: beyond propofol sedation during GI endoscopy. Dig Dis Sci.

[3] Bhananker SM, Posner KL, Cheney FW, Caplan RA, Lee LA, Domino KB (2006). Injury and liability associated with monitored anesthesia care: a closed claims analysis. Anesthesiology.

[4] Horiuchi A, Nakayama Y, Kajiyama M, Kato N, Kamijima T, Ichise Y, Tanaka N (2012). Safety and effectiveness of propofol sedation during and after outpatient colonoscopy. World J Gastroenterol.

[5] Luks AM, Swenson ER, Bärtsch P (2017). Acute high-altitude sickness. Eur Respir Rev.

[6] Lodeserto FJ, Lettich TM, Rezaie SR (2018). High-flow nasal cannula: mechanisms of action and adult and pediatric indications. Cureus.

[7] Wettstein RB, Shelledy DC, Peters JI (2005). Delivered oxygen concentrations using low-flow and high-flow nasal cannulas. Respir Care.

[8] Chikata Y, Onodera M, Oto J, Nishimura M (2017). FiO_2_ in an adult model simulating high-flow nasal cannula therapy. Respir Care.

[9] Ritchie JE, Williams AB, Gerard C, Hockey H (2011). Evaluation of a humidified nasal high-flow oxygen system, using oxygraphy, capnography and measurement of upper airway pressures. Anaesth Intensive Care.

[10] Vetrugno L, Deana C, Colaianni-Alfonso N, Tritapepe F, Fierro C, Maggiore SM (2024). Noninvasive respiratory support in the perioperative setting: a narrative review. Front Med.

[11] Frat JP, Thille AW, Mercat A, Girault C, Ragot S, Perbet S, Prat G, Boulain T, Morawiec E, Cottereau A, Devaquet J, Nseir S, Razazi K, Mira JP, Argaud L, Chakarian JC, Ricard JD, Wittebole X, Chevalier S, Herbland A, Fartoukh M, Constantin JM, Tonnelier JM, Pierrot M, Mathonnet A, Béduneau G, Delétage-Métreau C, Richard JC, Brochard L, Robert R (2015). FLORALI Study Group; REVA Network. High-flow oxygen through nasal cannula in acute hypoxemic respiratoryfailure. N Engl J Med.

[12] Azoulay E, Lemiale V, Mokart D, Nseir S, Argaud L, Pène F, Kontar L, Bruneel F, Klouche K, Barbier F, Reignier J, Berrahil-Meksen L, Louis G, Constantin JM, Mayaux J, Wallet F, Kouatchet A, Peigne V, Théodose I, Perez P, Girault C, Jaber S, Oziel J, Nyunga M, Terzi N, Bouadma L, Lebert C, Lautrette A, Bigé N, Raphalen JH, Papazian L, Darmon M, Chevret S, Demoule A (2018). Effect of high-flow nasal oxygen vs standard oxygen on 28-day mortality in immunocompromised patients with acute respiratory failure: the high randomized clinical trial. JAMA.

[13] Vetrugno L, Castaldo N, Fantin A, Deana C, Cortegiani A, Longhini F, Forfori F, Cammarota G, Grieco DL, Isola M, Navalesi P, Maggiore SM, Bassetti M, Chetta A, Confalonieri M, De Martino M, Ferrari G, Francisi D, Luzzati R, Meini S, Scozzafava M, Sozio E, Tascini C, Bassi F, Patruno V, Italian COVI-MIX, De Robertis E, Aldieri C, Ball L, Baratella E, Bartoletti M, Boscolo A, Burgazzi B, Catalanotti V, Confalonieri P, Corcione S, De Rosa FG, De Simoni A, Bono VD, Tria RD, Forlani S, Giacobbe DR, Granozzi B, Labate L, Lococo S, Lupia T, Matellon C, Mehrabi S, Morosi S, Mongodi S, Mura M, Nava S, Pol R, Pettenuzzo T, Quyen NH, Rescigno C, Righi E, Ruaro B, Salton F, Scabini S, Scarda A, Sibani M, Tacconelli E, Tartaglione G, Tazza B, Vania E, Viale P, Vianello A, Visentin A, Zuccon U, Meroi F, Buonsenso D, Study Group (2023). Ventilatory associated barotrauma in COVID-19 patients: a multicenter observational case control study (COVI-MIX-study). Pulmonology.

[14] Apfelbaum JL, Hagberg CA, Connis RT, Abdelmalak BB, Agarkar M, Dutton RP, Fiadjoe JE, Greif R, Klock PA, Mercier D, Myatra SN, O’Sullivan EP, Rosenblatt WH, Sorbello M, Tung A (2022). 2022 American society of anesthesiologists practice guidelines for management of the difficult airway. Anesthesiology.

[15] Groves N, Tobin A (2007). High flow nasal oxygen generates positive airway pressure in adult volunteers. Aust Crit Care.

[16] Mazzeffi MA, Petrick KM, Magder L, Greenwald BD, Darwin P, Goldberg EM, Bigeleisen P, Chow JH, Anders M, Boyd CM, Kaplowitz JS, Sun K, Terrin M, Rock P (2021). High-flow nasal cannula oxygen in patients having anesthesia for advanced esophagogastroduodenoscopy: HIFLOW-ENDO, a randomized clinical trial. Anesth Analg.

[17] Lin Y, Zhang X, Li L, Wei M, Zhao B, Wang X, Pan Z, Tian J, Yu W, Su D (2019). High-flow nasal cannula oxygen therapy and hypoxia during gastroscopy with propofol sedation: a randomized multicenter clinical trial. Gastrointest Endosc.

[18] Chernik DA, Gillings D, Laine H, Hendler J, Silver JM, Davidson AB, Schwam EM, Siegel JL (1990). Validity and reliability of the observer’s assessment of alertness/sedation scale: study with intravenous midazolam. J Clin Psychopharmacol.

[19] Mason KP, Green SM, Piacevoli Q, International Sedation Task Force (2012). Adverse event reporting tool to standardize the reporting and tracking of adverse events during procedural sedation: a consensus document from the World SIVA International Sedation Task Force. Br J Anaesth.

[20] Jiang B, Li Y, Ciren D, Dawa O, Feng Y, Laba C (2022). Supraglottic jet oxygenation and ventilation decreased hypoxemia during gastrointestinal endoscopy under deep sedation at high altitudes: a randomized clinical trial. BMC Anesthesiol.

[21] Santos-Martínez LE, Gómez-Tejada RA, Murillo-Jauregui CX, Hoyos-Paladines RA, Poyares-Jardim CV, Orozco-Levi M (2022). Chronic exposure to altitude. Clinical characteristics and diagnosis. Arch Cardiol Mex.

[22] Mauri T, Turrini C, Eronia N, Grasselli G, Volta CA, Bellani G, Pesenti A (2017). Physiologic effects of high-flow nasal cannula in acute hypoxemic respiratory failure. Am J Respir Crit Care Med.

[23] Parke RL, Eccleston ML, McGuinness SP (2011). The effects of flow on airway pressure during nasal high-flow oxygen therapy. Respir Care.

[24] Daniel MF, Mario GD, Edgar B, Mario V, Alejandra H, Nicolas G, Pablo V, Victor N, Albert V, Diego G, Antonio VS, Ramon MJ (2022). Use of high-flow nasal cannula in patients with pneumonia and hypoxemic respiratory failure at altitudes above 2600 m: what is the best predictor of success?. J Intensive Care Med.

